# Chronic allergy signaling: is it all stressed-out mitochondria?

**DOI:** 10.12703/r/11-37

**Published:** 2022-12-15

**Authors:** Syed-Rehan A Hussain, Mitchell H Grayson

**Affiliations:** 1 Division of Allergy and Immunology, Department of Pediatrics, Nationwide Children’s Hospital - The Ohio State University College of Medicine, Columbus, OH, USA; 2 Center for Clinical and Translational Research, Abigail Wexner Research Institute at Nationwide Children’s Hospital, Columbus, OH, USA

**Keywords:** metabolism, mitochondrial dysfunction, microRNA, obesity, homeostasis

## Abstract

Allergic diseases in general, and chronic allergic inflammation in particular, are on the rise in the United States and other developed countries. The idea of chronic allergic disease as a chronic type 2 immune response has been around for several decades. However, data suggest that other mechanisms may be important in chronic disease. Therefore, we believe it is time for a paradigm shift in understanding the mechanistic causes of disease symptoms in these diseases. In this review, we have avoided the classic canonical pathways and focused on the emerging idea that oxidative stress, changes in immuno-metabolism, mitochondrial dysfunction, and epigenetic changes (particularly microRNA profile) may be working concurrently or synergistically to potentiate allergic disease symptoms. Furthermore, we have addressed how the epidemic of obesity exacerbates allergic disease via the dysregulation of the aforementioned factors.

## Background

Allergy may be defined as an abnormal immune response to innocuous environmental substances (allergens), and the term was coined by acclaimed pediatrician Clemens von Pirquet in the early 20th century^1^. Potentially owing to less exposure to parasites and pathogenic and non-pathogenic bacteria skewing the development of normal immune response from type 1 helper (Th1) toward Th2 and, therefore, more exposure, particularly in urban populations, to non-infectious environmental allergens predisposing individuals to develop Th2 type cellular responses, allergic diseases are on the rise in developed countries^2^. The main types of allergens include fecal particles of house dust mites, animal dander, certain foods (e.g., peanuts, shellfish, milk, and eggs), certain medicines, and insect venoms.

Chronic or perennial allergies can occur at any time of the year. The common cause for chronic allergies is continuous or repetitive exposure, usually to indoor allergens, such as house dust mites, leading to persistent inflammation and causing changes in structural cells at the affected site, like airway epithelium and altered organ function. The structural changes and remodeling in chronic allergic inflammation are due to infiltration at the target site of several types of both innate and adaptive immune cells^2,3^. Unlike early- and late-phase allergic reactions from acute exposures that can be experimentally studied in human subjects, there is no clear understanding of the mechanism(s) involved in the development of chronic allergic inflammation.

In this review, rather than focusing on chronic Th2 cytokine (interleukin 4 [IL-4] and IL-13, in particular) pathway production, we propose that there are signaling pathways in mucosal cells induced by the stress of exposure to chronic allergies, and, thus, altered homeostasis leads to changes in immuno-metabolism and mitochondrial function. These are the proposed changes involving microRNAs (miRNAs) that drive the signaling pathways seen in chronic allergic disease and may even link chronic diseases like obesity with chronic allergic disease.

## Metabolism and homeostasis in chronic allergy

Although the knowledge about the influence of metabolic changes in allergic diseases that lead to exacerbation due to exposure to innocuous antigens is still being gathered, there is evidence that metabolism plays an important role in immune homeostasis^4–6^. Among the major mediators are reactive oxygen species (ROS), which are produced as a result of metabolic activity. The epithelial mucosal barrier is regulated by metabolism, as shown by the role of ROS in antimicrobial immunity. However, unchecked ROS production can cause damage and inflammatory lung diseases^7,8^. Similarly, IL-13 produced during an allergic reaction has been shown to increase ROS, and increased ROS can cause damage to mitochondria^3^. Studies have shown that in allergic disorders, the immunomodulatory and inflammatory properties of antigen-presenting cells are impacted by altered metabolism^3,9^. A 2017 murine study showed that the mechanistic target of rapamycin (mTOR) was required to regulate cellular metabolism by stimulating protein synthesis, glycolysis, lipid synthesis, and mitochondrial functions in dendritic cells and also influenced the immunologic character of allergic inflammation by skewing it toward a Th17/neutrophilic phenotype^10^. Furthermore, extracellular adenosine triphosphate (ATP), an important mediator of an inflammatory immune response, is present in high levels in patients with asthma^11^. ATP has been shown to induce Th17-mediated neutrophilic asthma in a mouse model^12^. So, it is conceivable that mitochondrial dysfunction (via increased ROS or perturbation of mTOR signaling or both) could lead to abnormally high ATP levels and a Th17/neutrophilic phenotype, which is associated with a more severe steroid-resistant type of disease, as we have recently shown^13^.

ROS, the by-product of aerobic metabolism, has an interesting dynamic, playing a role in both normal physiological functions and pathological conditions. Low levels of elevated ROS activate redox signaling pathways for normal physiological functions and immuno-biological responses^14–16^. However, oxidative stress causes high levels of ROS production, which is damaging to the system and has been shown to be involved in multiple pathological conditions^17,18^. Mitochondria are the major source of ROS production in cells. Chronic ROS production can cause oxidative damage to mitochondrial membrane proteins leading to the collapse of mitochondrial membrane potential (ΔΨm), affecting mitochondrial function, including ATP generation^19^. A collapse of ΔΨm is a standard indicator of mitochondrial damage, as reported by our group^20^ and others^18^.

## Mitochondria and signaling in allergy

Various environmental exposures, such as aeroallergens, can increase oxidative stress and ROS production, damage the electron transport chain (ETC), and dysregulate the expression of ETC genes that contribute to allergic inflammation^21,22^. Recent reports using cohorts from the Study of Asthma Phenotypes and Pharmacogenomic Interactions by Race-ethnicity (SAPPHIRE) and the Study of African Americans, Asthma, Genes & Environment II (SAGE II) have shown that patients with asthma had increased mitochondrial copy number in the peripheral blood; however, disease severity was not related to mitochondrial copy numbers, suggesting that high mitochondrial copy number may be intrinsic to asthma. Although there is no clear reason why the mitochondrial copy number is high in patients with asthma, it is hypothesized that high copy numbers indicate mitochondrial dysfunction as oxidative stress causes both damage to the mitochondria and upregulation of transcriptional and replication machinery to increase mitochondrial biogenesis^23^. Asthma was also associated with reduced expression of ETC genes affecting mitochondrial functions and pathways that are the critical final step in oxidative metabolism^24^. Thus, it can be suggested that mitochondrial copy number and the associated effect on ETC and ROS levels may provide subtle markers for specific treatment strategies, such as the use of antioxidants. However, several clinical trials are needed to delineate the impact of using antioxidants as an adjuvant to conventional therapy.

Cellular metabolism is impacted by mitochondria as they work in symbiosis with other cellular compartments to regulate metabolic processes, such as oxidative phosphorylation (OXPHOS) and fatty acid beta-oxidation. Furthermore, mitochondrial ROS (mtROS) causes calcium accumulation and affects the regulation of cell signaling, such as through nuclear factor kappa B (NF-κB) signaling^25,26^. Mitochondria also are actively involved in the FcεRI-dependent activation of mast cells by mtROS generation^27^. Furthermore, it has been shown in animal models that pre-existing mitochondrial dysfunction induced by environmental exposures, such as ragweed pollen, induces oxidative damage in mitochondria, causing an exacerbation of allergic inflammation upon re-exposure to the allergen. The hypersensitive response was associated with nuclear DNA-encoded mitochondrial proteins being damaged by ROS^21^. Similarly, cockroach allergen-induced oxidative stress produced mtROS in human bronchial epithelial cells, with cyclooxygenase-2 (COX-2) being significantly upregulated. This study demonstrated evidence of epigenetic regulation of COX-2 by showing a direct effect of miR-155, a small non-coding miRNA, in increasing the expression of COX-2^28^. These findings further suggest an interplay of epigenetic modulators, allergens, and mitochondria in allergic inflammation and highlight the importance of miRNAs in this process.

## MicroRNA control of epigenetics in allergic mechanism

Most epigenetic studies focus on the DNA methylation of genes^29–31^. Although our review is focused on chronic allergenic stimulation, it is worth noting that a small birth cohort study followed over a decade showed an epigenetic signature that linked to allergic disease development^32,33^. However, the data supporting the influence of methylation on allergic phenotype are somewhat opaque and only provide associations rather than demonstrating epigenetics as a predictive marker of allergic disease^32^. The lack of clarity in the findings is potentially due to the difficulty in collecting data from heterogenous groups and the small numbers of cases and controls.

miRNAs are the epigenetic regulators of various genes, including those involved in DNA methylation, and there is increasing evidence that circulating miRNAs, secreted by various tissues, including adipose tissue (AT), can impact metabolism; in fact, individuals with obesity have been shown to have an miRNA profile different from that of lean persons^34,35^. Furthermore, the nasal mucosa of patients with chronic asthma has been shown to have dysregulation of several miRNAs, including miR155, which is the same miRNA that was reported to be involved in the regulation of COX-2 and mtROS in the mitochondria of bronchial epithelial cells in patients with asthma^28,36^.

Therefore, unlike in DNA methylation profiling, to assess the effect on allergic diseases, it is much more feasible to use the circulating miRNA profile as a biomarker as miRNAs may be a more sensitive marker of chronic inflammation than DNA methylation^37^. In addition, circulating miRNAs may be a biomarker for obesity^38^. Thus, the miRNA biomarker profile can be associated with allergic diseases and is potentially a target for future therapeutic interventions.

## Obesity and chronic allergy

In addition to chronic stress due to allergen exposure, an area of research related to chronic allergic signaling has been the role of obesity in allergic disease. Obesity has reached epidemic levels in the United States and is a co-morbidity for severe chronic allergic diseases, presumably through dysregulation of cell metabolism. The innate components of allergic inflammation are alternatively activated macrophages (AAMs) (M2-type macrophages), ILC2, eosinophils, and regulatory T (Treg) cells. Homeostatic immune cell balance in a lean individual is anti-inflammatory, while obesity tilts toward a pro-inflammatory state with increased AAM, eosinophils, and ILC2 in visceral AT, a site where there is an interplay of cells and molecules that control metabolism and immunity^39,40^. It has been assumed that this increase in pro-atopic cell types connects obesity to allergic disease, and an excessive intake of saturated fatty acids (SFAs) has been shown to activate a pro-inflammatory phenotype through increased maturation, activation, and T-cell stimulation by dendritic cells^3^. So, obesity, which is a chronic health condition, has the ability to increase pro-atopic cells in the body while promoting a pro-inflammatory state (such as increased AAM, eosinophils, and ILC2 cells)^39^. But it may be more than just these changes that link obesity with atopic disease.

In obesity, there is potentially a bidirectional interplay between epigenetics and an imbalance in energy metabolism, and nutrients and lifestyle play major roles in the regulation of epigenetic mechanisms^41^. Adiposity, the impairment of the function of AT (particularly white AT, further subclassified as subcutaneous and visceral AT) contributes to obesity^41^. A 2017 study demonstrated that overfeeding of SFA led to a distinct DNA-methylation pattern in the AT of human subjects^42^.

**Figure 1.**
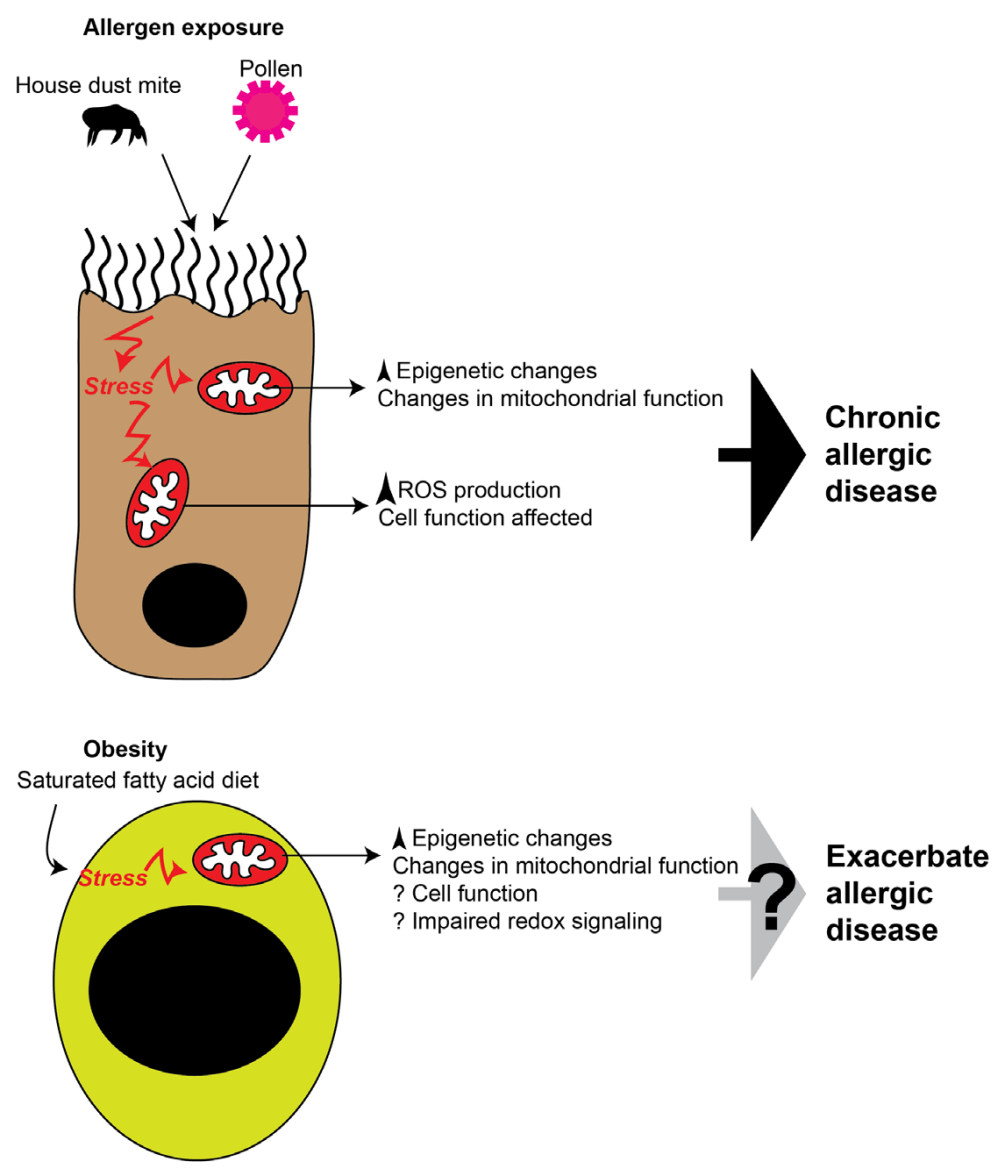
Potential role of mitochondrial stress in chronic allergic disease. The cartoon demonstrates the impact of allergen exposure on airway epithelial cell mitochondria (top panel) and subsequent mitochondrial dysfunction that may drive symptoms of chronic allergic disease. The bottom panel shows that obesity through a high saturated fatty acid diet drives similar stress responses in the mitochondria of macrophages and other phagocytic cells, and this may lead to the exacerbation of allergic diseases. ROS, reactive oxygen species.

miRNA expression is also considered either an epigenetic mechanism or a regulator of epigenetic machinery resulting in histone modification or DNA methylation^43^. Reports of miRNAs in mitochondria suggest that they regulate mitochondrial functions and adipogenesis, perhaps linking high caloric intake with impaired mitochondrial biogenesis and function^44^. The idea that this dysregulated mitochondrial function is associated with chronic disease is supported by the finding that miRNA profiles in obese individuals are different from those in lean individuals. Whether this difference is found in those with chronic allergic disease stimulation remains to be determined. Moreover, mitochondrial dysfunction can drive premature cell senescence and impaired mitophagy because of increased ROS production, and there is some evidence that it may influence allergy and asthma; furthermore, in obesity, there is an increase in cellular senescence^45,46^. Therefore, it can be argued that, in obesity, impaired mitophagy also may be contributing to the exacerbation of chronic allergy. It is intriguing to propose that chronic stress on mucosal epithelial cells could lead to impaired mitochondrial function, including increased epigenetic changes and impaired redox signaling (elevated ROS) impacting ATP levels, and these chronic “signals” drive allergic disease (Figure 1). As mentioned above, obesity drives similar stress, and these mitochondria-specific changes could be why obesity has a chronic Th2-like phenotype.

## Conclusions

We have tried to use “out of the box” thinking in this review to suggest that a major unmet area of research is the role of the mitochondria and miRNA in chronic allergic disease. Indeed, the idea that “stress” on the cells could be leading to the symptoms seen in these chronic diseases is not well explored. The fact that another disease (obesity) has been associated with similar metabolic and signaling changes provides additional support to this idea. Of course, it is still worth mentioning that chronic exposure to allergens, nonetheless will drive a chronic type 2 immune response (including elevated IL-4/13 and the downstream effects of mast cell activation). However, those pathways have been (and are being) well studied. Exploring the role of mitochondrial function and stress thus is a novel approach aspiring for further exploration.
